# Project Inclusive Genetics: Exploring the impact of patient-centered counseling training on physical disability bias in the prenatal setting

**DOI:** 10.1371/journal.pone.0255722

**Published:** 2021-08-05

**Authors:** Emma Vaimberg, Lindsay Demers, Eric Ford, Maya Sabatello, Blair Stevens, Shoumita Dasgupta

**Affiliations:** 1 Department of Pediatrics, Johns Hopkins Hospital, Baltimore, MD, United States of America; 2 Department of Medicine, Boston University School of Medicine, Boston, MA, United States of America; 3 Independent Scholar, Brooklyn, NY, United States of America; 4 Department of Medicine, Columbia University, New York, NY, United States of America; 5 Department of Medical Humanities and Ethics, Columbia University, New York, NY, United States of America; 6 Department of Obstetrics, Gynecology, and Reproductive Sciences, McGovern Medical School at UTHealth, Houston, TX, United States of America; 7 Department of Medicine, Biomedical Genetics Section, Boston University School of Medicine, Boston, MA, United States of America; Faculty of Medicine, National University of Mexico (UNAM), MEXICO

## Abstract

**Purpose:**

There is robust research examining the negative impact of racial and socioeconomic implicit bias on healthcare provider clinical decision-making. However, other under-studied important biases are likely to impact clinical care as well. The goal of this study was to explore the presence of bias against people with physical disability among a heterogeneous group of healthcare workers and trainees and to evaluate the effect of implicit association testing and an educational module on this bias.

**Method:**

The study was composed of a one-hour web-based survey and educational module. The survey included an explicit disability bias assessment, disability Implicit Association Tests (IATs), demographic collection, and pre- and post- module clinical vignettes of prenatal patient scenarios. In addition to providing counseling to hypothetical patients, participants also indicated their personal preferences on genetic testing and termination. The educational module focused on the principles of patient-centered counseling.

**Results:**

The collected data reflects responses from 335 participants. Within this sample, there were both explicit and implicit biases towards individuals with physical disabilities. Prior to the IAT and educational module, when respondents were tasked with providing genetic testing recommendations, implicit biases and personal preferences for genetic testing and termination influenced respondents’ clinical recommendations. Importantly, having previous professional experience with individuals with disabilities diminished biased clinical recommendations prior to the intervention. In response to the IAT and educational intervention, the effect of implicit bias and personal preferences on clinical recommendations decreased.

**Conclusions:**

This study demonstrates how bias against a marginalized group exists within the medical community and that personal opinions can impact clinical counseling. Importantly, our findings suggest that there are strategies that can be easily implemented into curricula to address disability bias, including formal educational interventions and the addition of professional experiences into healthcare professional training programs.

## Introduction

Numerous studies have demonstrated that unconscious, or implicit, bias held by healthcare providers can strongly and negatively impact patient encounters. This phenomenon has been observed in the context of racial bias, in which provider bias contributed to false beliefs about race-based biological differences in pain perception [1], misdiagnosis rates by patient race [2], worse healthcare outcomes [3], and poor interpersonal communication with Black patients [4]. Importantly, previous studies examining racial and socioeconomic biases among students have shown effective educational interventions that decrease implicit association test scores, indicating a decrease in implicit bias [5, 6]. Given these findings, it is possible that biases generally have the potential to contribute to healthcare disparities and may be amenable to educational intervention.

A small number of studies have explored healthcare providers’ and trainees’ attitudes toward people with disability and how education on disability and bias could affect those dispositions [7–10]. Many of these studies concluded that healthcare workers can have an improved outlook on people with disabilities after an educational intervention. However, bias in many of these studies was measured using an explicit bias approach, requiring self-reporting. Implicit Association Tests (IATs) have become the tool-of-choice for measuring bias because they can identify unreported, unconscious bias [11].

In our study, we sought to explore the existence of implicit disability bias among healthcare professionals and trainees, how this bias could impact genetic counseling recommendations in the prenatal setting, and whether this bias could be attenuated with an educational intervention and IAT. In addition to the clinician’s recognition of his or her bias, the clinician must understand the patient’s own bias, values, and needs. This understanding of both perspectives can lead to a shared decision-making model of clinical care. Our study incorporates many elements of a suggested framework for integrating implicit bias and shared decision-making education into health professionals’ curricula by increasing awareness of one’s own bias, emphasizing that bias impacts patient outcomes, and providing a patient-centered counseling model [12–14]. The full study explores both physical and intellectual disability using two different, validated IATs. However, we focus here on the findings from the physical disability (PD) section in order to robustly discuss the breadth of results.

## Methods

### Study design

**Fig 1:**
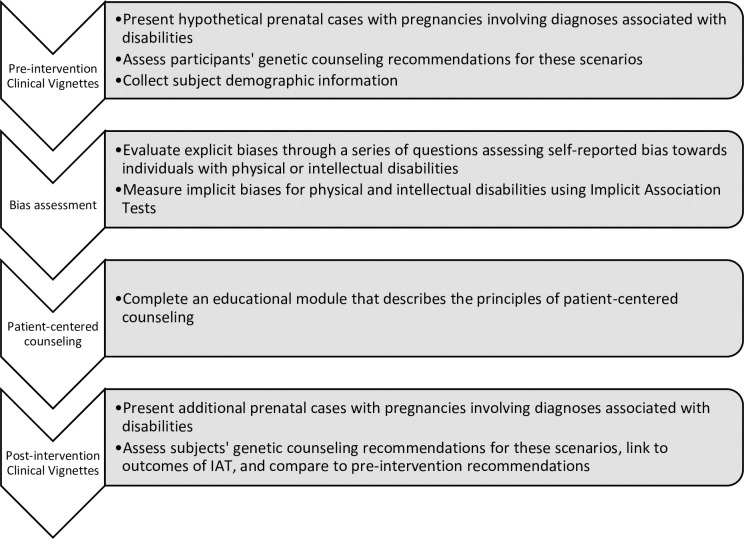
Graphical representation of experimental design. Participants were presented with pre-intervention clinical scenarios, questions assessing explicit bias, implicit bias testing, a novel educational module on the principles of patient-centered counseling, and post-intervention clinical scenarios. See S1 Appendix for the complete module.

Our web-based study included an initial set of clinical scenarios with survey questions, a demographic questionnaire, a physical disability IAT [15], our novel interactive educational module, and a second set of clinical scenarios with a survey. This study was designed to include both the IAT and the educational module to assess both pre-existing biases and changes in participants’ responses to clinical scenarios. Participants could receive continuing medical education (CME) credit or continuing education units (CEUs) for their participation in our study. Those requesting continuing education credit were given an additional multiple-choice test on the contents of the teaching section (Fig 1). The complete module is available as S1 Appendix.

### Clinical scenarios and educational module

The clinical scenarios and educational module were generated by one of the authors, a genetic counselor with expertise in prenatal counseling [BS], with input from the full Project Inclusive Genetics Steering Committee and pilot group. Clinical cases involved a pregnancy with a significant chance of inheriting a genetic condition with a PD. Each condition was briefly described and was specifically chosen to have similar impacts on daily living. The physical disability conditions assessed in the survey were Becker’s Muscular Dystrophy (S1 Appendix, pages 3–4) and Ataxia Telangiectasia (S1 Appendix, pages 43–44).

Within each case, there were two scenarios: 1) a patient who would terminate an affected pregnancy and 2) a patient who would not terminate an affected pregnancy. Respondents were asked to rate on a scale of 1–5 how likely they were to encourage or discourage prenatal genetic testing and whether they would personally obtain genetic testing and/or seek termination for their own similar pregnancy.

### Outcome measures

For all scenarios, we examined the likelihood of a respondent to recommend genetic testing *and* whether that recommendation was an ethically appropriate response, as outlined by the American College of Obstetrics and Gynecology [16, 17] and the American College of Medical Genetics and Genomics [18, 19]. We further investigated what respondent-specific factors may impact the propensity to recommend testing and the likelihood of correctly recommending testing. These relationships were measured both before and after our intervention, IAT followed by the educational module.

In the instance when the patient would terminate an affected pregnancy with a PD-positive result, the correct counseling recommendation would be to offer and/or encourage prenatal genetic testing (answer choices 1, 2, and 3). Whether a healthcare provider *encourages* versus *offers* genetic testing when a patient indicates they are considering termination may reflect a provider’s personal bias. In the case when the patient would not terminate the pregnancy regardless of the outcome of prenatal testing, the appropriate counseling recommendation is to offer testing, but to leave the decision of pursuing testing to the patient. There are many reasons an expecting parent may wish to get prenatal testing outside of termination, including lining up specialists for postnatal care, preparing the family for a child with a disability, and making physical alterations to the home to accommodate a child with a PD.

### Pilot study

We piloted our study with eight members of the National Human Genome Research Institute’s Inter-Society Coordinating Committee for Practitioner Education in Genomics (ISCC) [20]. The pilot group provided feedback on the structure of the study and expertise in genetic testing and counseling. Revisions were made based on feedback, and subsequently official participant recruitment to our online module began (link to full module here: https://www.bu.edu/project-inclusive/).

### Participant recruitment

We recruited participants from diverse training backgrounds from January 11, 2020 to March 28, 2020. Given the broad distribution of the study and desire to collect data from a diverse set of participants, there were no specific inclusion/exclusion criteria aside from full completion of the module. Pre-clinical medical students were recruited at the Boston University School of Medicine (BUSM) who were enrolled in the Genomic Medicine course. This study was provided as an online pre-class assignment with the option to complete a non-data collecting version. All of the students opted to complete the study. Clinical medical students from BUSM were recruited through their third- and fourth-year clerkships as an optional, didactic activity. BUSM clerkship directors were provided the link to the study via email in the setting of cancellation of multiple medical student rotations due to the Covid-19 pandemic. All students were ensured that their grade was not linked to participation in the study, and participation was entirely voluntary. The website link to the study was also disseminated to genetic counselors through the National Society of Genetic Counselors, ISCC meetings, and professional networks. All other participants were recruited through an IRB-approved listserv email advertisement sent by the Boston University Continuing Medical Education Office. Providers who completed the module were eligible to receive free continuing education credits. There was no in-person portion to the study, and participants conducted the study from personal or professional computers.

### Ethics statement

All participants were ensured anonymity, and written consent was obtained prior to the start of the study. Our study was approved by the Boston University Institutional Review Board (H-38446).

### Software

The evaluation code was built using MinnoJS, a Javascript library for creating web-based questionnaires and reaction time tasks [21]. Our code is composed of both JSON inputs to MinnoJS and original Javascript code and is hosted on an independent web server. The inputs to MinnoJS describe the ordering of the module and the question text, types, answers, and descriptions.

Data were collected and saved to three comma-separated files. First, a record of all initial participants was kept to compare the number of participants who began the module and the number who finished. The second file contained IAT results. Finally, a complete set of data, including participant identifications, raw data from the IATs, and answers to all questionnaires were recorded.

These outputs were processed through a Python script that removed the raw IAT results and various meta-data, such as response and response latency times. At this point the identifying information was separated from the questionnaire and IAT results in order to provide anonymity to the participants. The resulting anonymized IAT results, demographic responses, and clinical scenario answers were collated and saved to a single comma-separated file for further data analysis. Identifying information was necessary to provide continuing education credits, so a firewall was created using a separate Python script that gathered only identifying information and continuing education quiz scores.

### Statistical analysis

All statistical analyses were performed using SPSS, and study data (S2 and S3 Appendices) reflects the participant recruitment and data collection period of January 11, 2020 to March 28, 2020.

We collected respondents’ demographic data, including personal contact—either prior first-hand or professional experiences—with individuals with disability. From these responses, we assigned a score of 0 (no experience) or 1 (one or more experiences). First-hand experience included personal history of disability and a family member or friend with a disability. Professional experience included volunteerism, clinical experiences, and “other.”

The explicit bias assessment consisted of five items reflecting negative societal attitudes (e.g., “most people are uncomfortable around a child with a physical disability”). These items were selected and adapted from other studies to focus on this study’s population [22, 23]. The explicit bias score was calculated as a fraction of possible “explicit bias points” divided by 25, the maximum number of points derived from the explicit bias questions (S1 Appendix, page 18). This generated a single explicit bias score per respondent that ranged from 0.2 to 1.00, with a higher number indicating more bias. Implicit bias scoring has been previously described [21, 24].

All bivariate relationships were estimated using Spearman’s rho. To determine the impact that multiple variables had on clinical decision-making, we used a series of logistic regressions, and non-parametric testing to compare pre- and post-educational module decision-making.

## Results

Overall, 335 respondents completed the study. A complete demographic profile of the study subjects can be found in Table 1. To summarize our cohort briefly, the majority of the participants were female, there was a predominance of genetic counselors and pre-clinical medical students, and most of the participants had at least one professional experience with individuals with physical disability. Additionally, greater than 87% of the participants were very or somewhat Pro-Choice.

**Table 1:** Respondent Demographic data, total respondents = 335.

	N (%)
**Gender**	
Male	86 (25.7)
Female	241 (71.9)
Non-binary/prefer not to answer	3 (0.9)
Did not answer	5 (1.5)
**Clinical Setting**	
Medical Doctor (MD)	11 (3.3)
Genetic Counselor (GC)	123 (36.7)
Nurse	14 (4.2)
Pre-clinical MD student^a^	127 (37.9)
Clinical MD student	27 (8.1)
GC student	5 (1.5)
Other	25 (7.5)
Did not answer	3 (0.9)
**Prior Experience**	
First-Hand Experience^b^	164 (49)
Professional Experience^c^	277 (82.7)
**Opinions on Termination**	
Very Pro-Choice	215 (64.2)
Somewhat Pro-Choice	78 (23.3)
Neutral	11 (3.3)
Somewhat Pro-Life	17 (5.1)
Very Pro-Life	4 (1.2)
Did not answer	10 (3.0)

^a^ Pre-clinical MD student is a student who has not yet begun clerkships or rotations in the hospital. The student may have had clinical contact in the context of shadowing or training on patient interviewing skills.

^b^ First-hand experience is defined as having a personal history of disability or having a family member or friend with a disability

^c^ Professional experience is defined as having had a volunteer experience, clinical experience, or other.

### Implicit and explicit bias measures

**Fig 2:**
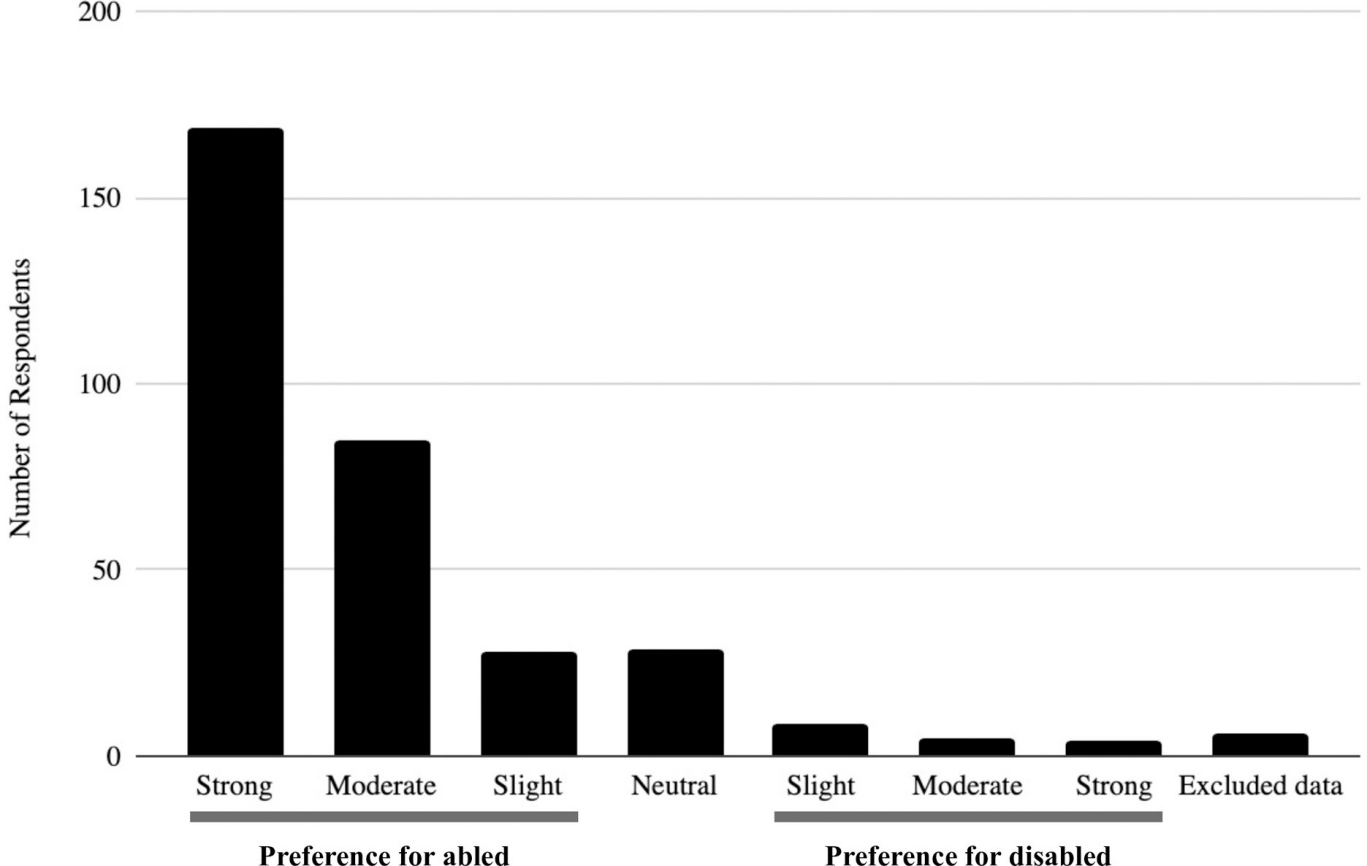
Results of physical disability implicit association test from 335 respondents.

We measured both explicit and implicit biases against individuals with PD in our cohort. The average explicit bias was 0.615 ± 0.105, indicating a slight explicit preference for abled individuals, and 84% of respondents had an implicit preference for physically-abled individuals (Fig 2). There was no significant relationship between the existence of an explicit and implicit bias (r = - 0.014, p = 0.808).

### Factors that impact the likelihood of recommending genetic testing

We next examined factors associated with the likelihood of recommending prenatal testing to patients using Spearman correlations (Table 2). Explicit bias did not significantly impact clinical recommendations (all p > 0.05). However, respondents with greater implicit biases against people with PD were more likely to recommend prenatal genetic testing to patients who would terminate a PD-positive pregnancy (r = - 0.127, p = 0.021), suggesting an influence of unconscious bias on respondents’ clinical recommendations prior to the module and IAT. After these interventions, this relationship between implicit bias and the propensity to recommend genetic testing was reduced and no longer significant (r = - 0.042, p = 0.446).

**Table 2:** Spearman correlations for the likelihood of recommending genetic testing pre- and post- intervention in cases where patients would (a) and would not (b) terminate an affected pregnancy.

**a. Patient would terminate**		
	**Spearman r**	**Significance (p)**
**Explicit Bias**		
Pre-module	- 0.068	.248
Post-module	- 0.016	0.785
**Implicit Bias**		
Pre-module	**- 0.127**	**.021**
Post-module	- 0.042	0.446
**Personally would test**		
pre-module	**0.290**	**< 0.001**
post-module	**0.263**	**< 0.001**
**Personally would terminate**		
Pre-module	**0.301**	**< 0.001**
Post-module	**0.206**	**< 0.001**
**b. patient would not terminate**		
**Explicit Bias**		
Pre-module	- 0.074	0.207
Post-module	- 0.018	0.754
**Implicit Bias**		
Pre-module	- 0.076	0.169
Post-module	0.014	0.798
**Personally would test**		
pre-module	**0.219**	**< 0.001**
post-module	0.095	0.082
**Personally would terminate**		
Pre-module	0.077	0.162
Post-module	- 0.018	0.739

Bolded text indicates significant results. N = 335 participants.

We further divided our cohort into two sub-groups, biased against people with PD (n = 282) and biased for people with PD/neutral preference (n = 47) to examine the impact of the intervention on clinical recommendations based on provider bias. S1 Table shows the primary data. A negative difference is when pre-intervention, the participant would recommend genetic testing, but post-intervention would no longer recommend testing. A positive difference reflects a change where pre-intervention, the participant would not recommend genetic testing, but post-intervention would recommend testing. No change means that the participant did not change their response; this includes both would and would not recommend testing. Using this primary data, we compared clinical recommendations pre- and post-intervention using non-parametric sign tests and found that the module and IAT together had a greater, statistically significant impact on the group biased against people with PD; the propensity to recommend testing after the intervention was decreased in both termination and non-termination scenarios (Z (1) = -2.507, p = 0.012 and Z (1) = -3.233, p = 0.001, respectively), as compared to the biased for disability/neutral group (p > .05 for both).

We also assessed whether personal preferences had any impact on clinical recommendations, as this could also reflect a bias that is not directly tested by IATs. We found that respondents who were more likely to obtain prenatal testing for their own pregnancy were also more likely to recommend prenatal testing to their patients, regardless of the patients’ attitudes towards termination (would terminate: r = 0.290, p <0.001; would not terminate: r = 0.219, p < 0.001).

Additionally, respondents who were more likely to terminate their own PD-positive pregnancy were more likely to recommend genetic testing to those patients who would also terminate a PD-positive pregnancy (r = 0.301, p < 0.001). However, respondents who reported they would terminate their own PD-positive pregnancy were not significantly more likely to recommend genetic testing to patients that did not intend to terminate a PD-positive pregnancy (p = 0.162).

After completing the IAT and educational module, the influence of provider personal preferences on genetic testing recommendations in the termination scenario persisted (testing: r = 0.263, p <0.001; termination: r = 0.206, p <0.001). However, the correlation between personal testing and recommending testing was no longer significant after completion of the educational module and IAT for patients who would not terminate their pregnancies (r = 0.095, p = 0.082).

We further explored the impact of various factors on clinical decision-making using logistic regression analysis, where we controlled for explicit and implicit bias scores, personal contact with people with PD, and respondent personal preferences (S2 Table). We continued to observe that prior to our intervention when the patient would terminate a PD-positive pregnancy, personal preference for termination and inclination to recommend genetic testing were still strongly linked (p = 0.001), again demonstrating that a provider’s preference for termination can independently predict when the provider would recommend genetic testing.

However, we also observed that some of the relationships previously identified were no longer significantly correlated with clinical decision-making when considered independently of one another. For example, the personal preference to get genetic testing was identified as a factor that increased the likelihood of recommending genetic testing by bivariate analysis but was no longer significantly correlated by logistic regression analysis. Disagreement between these two analyses likely reflects unmeasured factors in our experiment, such as a general preference for medical intervention and implicit bias not adequately measured by IAT.

Another relationship uncovered in the logistic regression analysis, not previously observed by bivariate analysis, is that after the intervention, those with an implicit bias against individuals with PDs were less likely to recommend genetic testing to patients who would not terminate a PD-positive pregnancy (p = 0.041, S2D Table).

Additionally, logistic regression analysis demonstrated an important relationship between personal contact with people with PDs and likelihood of testing. We found that having one or more personal contacts in a professional setting can significantly decrease the likelihood of recommending testing in some clinical situations (S2A, S2B and S2D Table), pointing to another factor that can influence clinical recommendations and suggesting that bias for medical intervention may be mitigated with specific clinical experiences.

### Participant factors that predict appropriate counseling recommendations

We next assessed whether there were any respondent-specific factors that could predict whether a respondent would *correctly* advise a patient on prenatal testing. For these analyses, recall that the correct counseling recommendations are to offer and/or encourage testing when a patient would terminate a PD-positive pregnancy and only to offer testing when a patient would not terminate a PD-positive pregnancy.

In the instance when a patient would terminate, 99.4% of respondents correctly offered and/or encouraged testing (see S2 and S3 Appendices). Given the homogeneity in responses, there was insufficient variability to perform additional analyses.

Therefore, we focused on the recommendations to the patient who would not terminate a PD-positive pregnancy. We again used a series of logistic regression analyses and found that factors that predicted whether a respondent was more likely to correctly offer but not encourage/discourage testing included having a history of professional contact with people with PD and being a genetics specialist (see Table 3). Interestingly, we found that having professional experience no longer predicted whether a participant would correctly advise patients in the non-termination scenario (Table 3B). We also observed after our interventions, participants with an implicit bias against PD were more likely to correctly advise hypothetical patients (Table 3B).

**Table 3:** Results from logistic regression analyses of factors that increase the likelihood of *correctly counseling* patients who would not terminate a PD-positive pregnancy.

**a. Pre-intervention**							
	B	S.E.	Wald	df	p	Exp(B)	Probability
First-hand Exp	-0.161	0.303	0.284	1	0.594	0.851	0.701
**Professional Exp**	**0.813**	**0.354**	**5.274**	**1**	**0.022**	**2.254**	**0.905**
Explicit Bias	-2.234	1.587	1.981	1	0.159	0.107	0.527
Implicit Bias	-0.159	0.139	1.321	1	0.25	0.853	0.701
personally would test	0.154	0.421	0.135	1	0.714	1.167	0.763
personally would terminate	-0.215	0.337	0.405	1	0.525	0.807	0.691
Genetics specialist^a^	0.716	0.375	3.634	1	0.057	2.045	0.885
Constant	2.882	1.4	4.239	1	0.04	17.853	1.000
Cox & Snell R^2^ = 0.064							
**b. Post-intervention**							
	B	S.E.	Wald	df	Sig.	Exp(B)	Probability
First-hand Exp	-0.417	0.341	1.494	1	0.222	0.659	0.659
Professional Exp	0.333	0.394	0.717	1	0.397	1.395	0.801
Explicit Bias	0.096	1.648	0.003	1	0.954	1.1	0.750
**Implicit Bias**	**0.264**	**0.133**	**3.944**	**1**	**0.047**	**1.302**	**0.786**
personally would test	-0.104	0.898	0.013	1	0.908	0.901	0.711
personally would terminate	0.234	0.449	0.272	1	0.602	1.264	0.780
**Genetics specialist**^**a**^	**1.94**	**0.561**	**11.969**	**1**	**0.001**	**6.958**	**0.999**
Constant	-0.547	1.567	0.122	1	0.727	0.579	0.641
Cox & Snell R^2^ = 0.088							

a. Pre-intervention: Factors that impact appropriate counseling *prior* to the educational intervention. Findings that are significant are indicated in bolded text.

b. Post-intervention: Factors that impact appropriate counseling *after* the educational intervention. Findings that are significant are indicated in bolded text.

^a^ Genetics specialist includes individuals who have received formal longitudinal training in patient-centered genetic counseling, such as genetic counselors and physician geneticists.

When we compared clinical recommendations pre- and post-module, we found that the intervention increased the number of correct clinical recommendations overall (Z (1) = - 2.165, p = 0.030). When we subdivided the groups into biased against people with PD (n = 282) and biased for people with PD/neutral preference (n = 47). S3 Table contains the absolute numbers of negative difference (genetic testing recommendation changes from correct to incorrect), positive difference (genetic testing recommendation changes from incorrect to correct), and no change. We examined the changes in these recommendations for both scenario 1 and 2. We found that our intervention was more effective on individuals in the group biased against PD (Z(1) = -3.123, p = 0.002), as compared to respondents who were biased for or neutral towards people with PD (p > 0.05), specifically in scenario 2, when the patient would not terminate the pregnancy.

## Discussion

### Summary of results

It is important to examine the role of bias in clinical care in order to promote equity and inclusion in the practice of medicine. In our study, we focused on bias against people with PD and whether that bias can impact clinical decision-making. Broadly speaking, we found that among healthcare workers both explicit and implicit biases exist and that the existence of implicit bias and personal preferences were associated with the increased likelihood to recommend prenatal testing. Furthermore, we demonstrated that our module with an IAT can promote ethically appropriate decision-making and reduce the potential influence of personal biases on clinical recommendations, particularly on providers with a preconceived bias. Importantly, our findings suggest that recognition of the possibility of bias by taking an IAT, training in patient-centered counseling, and professional exposure to individuals with PD can have a positive effect on promoting patient-centered care. Altogether, these findings suggest the importance of education on patient-centered genetic counseling and increasing exposure to individuals with PDs.

There are some specific findings that we feel warrant further discussion. First, in assessing the likelihood of recommending genetic testing, we observed that the correlation between personal testing preferences and testing recommendations was not significant after the intervention in the non-termination scenario. Since diagnostic testing is not necessary, but optional, for patients who do not plan to terminate, genetic testing recommendations should be offered and not recommended. Therefore, these results do demonstrate the impact of our combined educational module/implicit bias testing intervention.

In contrast, in the termination scenario, we found that even after our interventions, provider personal preferences for termination continued to influence provider recommendations. However, in this case, one can argue that genetic testing should be encouraged since diagnostic testing is imperative for informed decision-making for the patients. Therefore, educational modules and implicit bias training may not have an impact on clinical recommendations to undergo testing in this particular clinical scenario.

Additionally, in our logistic regression analysis, we uncovered the impact of implicit bias on testing recommendations. In the non-termination scenario after the IAT and educational module, participants with an implicit bias against PD were less likely to offer genetic testing and more likely to correctly advise patients (S2D Table and Table 3B). These were fairly weak observations (p = 0.041 and p = 0.047, respectively), but may suggest that our interventions have a greater impact on those with a bias against PD—the group in which we previously demonstrated was more likely to recommend clinical testing in certain patient scenarios prior to our intervention. Of note, our goal is not to limit access to genetic testing, but rather to prompt providers to thoughtfully consider patient wishes prior to acting on their own belief systems.

Finally, in some, but not all, clinical scenarios, we found that after our intervention, having professional experiences did not always significantly decrease the likelihood of recommending testing or increase the chances of correctly advising a patient (S1C Table and Table 3B, respectively). We believe these findings may suggest that our intervention provides additional context for those who have not had professional experiences and may have the potential to be used as an additional method in training programs in which increasing professional exposure is not a readily available option.

### Discussion of methods for improving disability education

Overall, the findings in our study suggests a role for both formal learning and service learning in health professions education. Possible interventions to increase disability education among healthcare providers vary. The most common approach is a conventional lecture or seminar delivered by faculty, followed by supervised encounters with patients and advocates. However, these may not be the most effective interventions to promote disability-related knowledge [25]. Rather, positive changes are more likely to occur when a person with a disability acts as an expert to teach students, and students are provided with the opportunity to reflect on their own biases [26, 27].

The finding that our educational module along with an IAT—a relatively short intervention—can reduce stigma is promising. However, as in other interventions, it is not known whether its impact will be long-lasting. For medical programs to address disability biases among healthcare providers and students, trainings should promote self-reflection about disability biases and provide information about the needs, rights, and culture of people with disabilities [28]. Consistent reinforcement of these principles across the educational stages of students and healthcare professionals is likely to yield the best outcome [25].

The mitigating role of personal contact in a professional setting on disability bias further strengthens this suggestion for future intervention. As described in this study, participants who reported one or more professional experiences with people with disabilities had lower implicit bias scores and were more likely to provide the correct counseling recommendations. This finding extends previous research on stigma against people with psychiatric conditions [29–31] to those with PD and is important for two reasons. First, it demonstrates that healthcare providers, as members of the general public, have stigmatizing views about individuals with disabilities and that professional contact reduces stigma. Second, our findings highlight the importance of promoting interaction between healthcare providers and members of diverse and marginalized groups to reduce bias and improve clinical care.

### Limitations

Our study has several limitations. First, most of the practicing clinicians were trained genetic counselors (GCs). Given that GCs complete curricula dedicated to patient-centered counseling as part of their professional training, it may be that we did not observe the full impact that our module could have, particularly on physicians, nurse practitioners, and physician assistants whose training does not necessarily include genetic counseling.

Second, the differences in clinical and personal decision-making between clinical scenarios could, in part, be due to the genetic conditions assessed. While conditions were strategically selected to have the most similar phenotype and impact on daily living, respondents could have previous first-hand or professional experiences with a particular genetic condition that could further impact decision-making and recommendations.

Third, in our study we examined the impact of both an educational module on patient-centered counseling and an IAT on clinical decision-making. Due to the nature of the study, it is not clear whether one of these interventions had more of an impact than the other. Prior to entering the module, participants were presented with learning objectives that introduced the idea of implicit bias as a part of the study. They performed the IATs before completing the educational module and second set of clinical scenarios. IATs have been used in past studies as both a tool to measure bias and as an intervention to promote awareness of bias. In our study, it likely served this dual purpose despite the module design deferring IAT results in an attempt to focus primarily on the impact of the patient-centered counseling educational module. IAT results were not disclosed to the study participants until the end of the data collection portion of the study, thus for the IAT to impact results, it would have to be based on familiarity with or inference of what IATs are designed to measure. Overall, our goal for this study was to establish the existence of a bias that can impact clinical decision-making and to introduce possible ways to mitigate its effects. By administering the IAT prior to the educational module, it can be used as a tool to measure implicit bias. However, it remains unclear whether the IAT alone had an impact on clinical decision-making in the second set of clinical scenarios. Future work may be designed to include an IAT-only arm to allow independent assessment of the impact of the IAT and educational module.

Finally, our respondents were generally highly supportive of termination in almost any situation (Table 1). We did not have enough power in our sample to evaluate whether opinions opposing termination, e.g. Pro-Life or Anti-Choice views, could independently affect clinical decision-making. This final point applies more broadly in that it is not known whether our participants adequately represent the larger community of medical trainees, medical providers, GCs, and GC trainees. The preclinical student sample was representative of the Boston University School of Medicine first year medical student cohort in that all students opted into the study; however, other medical schools may differ from our student cohort at a private medical school in the Northeast. We aim to conduct future analyses to examine this specific question. For the clinical student cohort, there were only 27 participants. Again, we aim to incorporate more early learners that have clinical experience in future studies. Finally, among the providers, the most strongly represented group was genetic counselors, which is a relatively homogeneous group in training and demographics, so we anticipate that our sample would be representative. Further recruitment is planned to reach similar confidence with other health care providers including MDs, DOs, and nurses.

### Final remarks

Overall, our study demonstrates that personal opinions and biases against PD can impact clinical care, and dedicated instruction on implicit bias and patient-centered counseling can counteract these effects. As availability of genetic tests continues to grow, the ability of providers to advise patients in an inclusive, unbiased way becomes increasingly important. Future investigations should focus on a wider variety of healthcare providers as targeting a spectrum of healthcare providers could provide further insight into the impact of bias and formal education on clinical recommendations. The website remains open and freely available for interested readers to complete the module individually, as part of their residency training programs, and in other settings (link to study here: https://www.bu.edu/project-inclusive/).

## Supporting information

S1 Appendix Static version of consent, clinical vignettes, IATs, demographics questionnaire, and educational module. (PDF)

S2 Appendix De-identified dataset. (CSV)

S3 Appendix De-identified dataset. (XLSX)

S1 Table Input data for non-parametric sign test: Sub-grouping of biased for/neutral towards (a) and biased against (b) individuals with PD. Input data for changes in recommendations for genetic testing pre- and post- intervention in scenarios where patients would and would not terminate the pregnancy.
(DOCX)

S2 Table Results from logistic regression analyses pre (a, b) and post (c, d) educational module.
(DOCX)

S3 Table Input data for non-parametric sign test: Sub-grouping of biased for/neutral towards (a) and biased against (b) individuals with PD. Input data for changes in correctness of recommendations pre- and post- intervention in scenarios where patients would and would not terminate the pregnancy.
(DOCX)

## Abbreviations

IAT: Implicit Association Test
PD: Physical Disability
ISCC: Inter-Society Coordinating Committee for Practitioner Education in Genomics
BUSM: Boston University School of Medicine
MD: Medical Doctor
GC: Genetic Counselor
