# Supplementary material for: Project Inclusive Genetics: Exploring the impact of patient-centered counseling training on physical disability bias in the prenatal setting
Source: PLoS One. 2021 Aug 5;16(8):e0255722. doi: 10.1371/journal.pone.0255722 (PMC8341652; doi:10.1371/journal.pone.0255722)
Supplement: S1 Appendix — (PDF) [file pone.0255722.s001.pdf]

Clinical Decision-Making in the Prenatal Setting

Research consent form

Basic Information

Title of Project: Project Inclusive Genetics

IRB Number: H-38446

Sponsor: Association of American Medical Colleges

Principal Investigator:

Shoumita Dasgupta
dasgupta@bu.edu
72 East Concord Street, E-200
Boston, MA 02118

Study Phone Number: 617-358-7288

Overview/Purpose

We are asking you to be in a research study. We are doing the research to examine the clinical decision-making practices of providers and trainees in the prenatal setting. If you agree, you will complete a pre- and post-survey and review an educational module on the principles of patient-centered counseling and shared decision-making.

What Will Happen in This Research Study

The study will include hypothetic prenatal cases with multiple response choices, a survey of background information, an educational module involving elements of social psychology and principles of patient-centered counseling. All of these elements will be accessed on-line, and it is anticipated that the full series of activities will take 30–60 minutes to complete.

Risks and Benefits

There are no perceived physical risks or anticipated adverse effects resulting from your participation in this study.

There is no direct benefit to you from participation. Your being in the study may help the investigators learn about clinical decision-making practices.

Confidentiality

We must use information that shows your identity only for the purpose of providing CME/CEU credits, if you choose to receive them. The rest of the information that you provide in this study will be handled anonymously. Any information that is personally identifiable will not be included for response analysis.

Subject's Rights

By consenting to be in this study, you do not waive any of your legal rights. Consenting means that you have been given information about this study and that you agree to participate in the study. Please save this form or contact the study team if you would like a copy of this form to keep.

If you do not agree to be in this study or if at any time you withdraw from this study, you will not suffer any penalty or lose any benefits to which you are entitled. Your participation is completely up to you. Your decision will not affect your ability to get health care or payment for your health care. It will not affect your enrollment in any health plan or benefits you can get.

Questions

The investigator or a member of the research team will try to answer all of your questions. If you have questions or concerns at any time, contact Emma Vaimberg at vaimberg@bu.edu.

You may also call 617-358-5372 or email medirb@bu.edu. You will be talking to someone at the Boston Medical Center and Boston University Medical Campus IRB. The IRB is a group that helps monitor research. You should call or email the IRB if you want to find out about your rights as a research subject. You should also call or email if you want to talk to someone who is not part of the study about your questions, concerns, or problems.

By agreeing to be in this research and clicking Yes to participate, you are indicating that you have read this form (or it has been read to you), that your questions have been answered to your satisfaction, and that you voluntarily agree to participate in this research study.

Re-contact

We would like to ask your permission to contact you again in the future. This contact would be after the study has ended. Please select your choices below:

- You may contact me again to ask for additional information related to this study.
- You may contact me again to let me know about a different research study.

Participation

I have read the above information, have been provided with the opportunity to have any question about this study answered, and:

- **I agree to participate**
- I decline to participate

Continuing Ed credit

I may claim CME or CEU credits for this course now or in the future:

- **Yes**
- No

I may claim credit for:

CEU

Please enter your name:

Please enter your email address:

If you are claiming CEU credit, please enter you NSGC user ID. If you do not have an NSGC ID number enter "n/a".

Submit

Patient-Centered Genetic Counseling

Agenda

The participant will have the opportunity to engage in a professional development exercise focusing on clinical decision making in the prenatal setting. The module will use both hypothetical clinical prenatal cases and an educational component introducing the principles of patient-centered counseling and shared decision-making.

Needs Assessment

All health care providers (and students who will become providers) bring personal experience into the exam room. These experiences can sometimes make it difficult to apply patient-centered genetic counseling techniques in prenatal clinics. Even though there is a best counseling strategy, there is no instrument to determine the specific relationship between personal experiences and the genetic counseling recommendations by current providers. There is currently also a lack of training/curriculum offered to students (future health care professionals). This program aims to provide participants with the tools needed to follow best practices in patient-centered counseling.

Learning Objectives

After completing this online activity, learners will be able to:

1. Identify personal implicit biases towards individuals with physical and/or intellectual disability.
2. Illustrate principles of patient-centered counseling.
3. Apply patient-centered counseling approaches to cases involving patients with disabilities in order to promote patient-centered care.

Target Audience

The target audience is physicians, genetic counselors, other health care providers, faculty and students who are interested in counseling prenatal clinical genetic disorders. Health care providers need not specialize in genetics to benefit from this program.

BOSTON UNIVERSITY

Accreditation Statement

Boston University School of Medicine is accredited by the Accreditation Council for Continuing Medical Education to provide continuing medical education for physicians.

Boston University School of Medicine designates this enduring material for a maximum of 1.0 *AMA PRA Category 1 Credit*™. Participants should claim only the credit commensurate with the extent of their participation in the activity.

Genetic Counselor CEUs: This event has been submitted to the National Society of Genetic Counselors (NSGC) for approval of Category 1 CEUs. The American Board of Genetic Counseling (ABGC) accepts CEUs approved by NSGC for purposes of recertification. Approval for the requested CEUs and Contact Hours is currently pending.

Faculty Disclosures

Boston University School of Medicine asks all individuals, and their spouses/partners, involved in the development and presentation of Continuing Medical Education (CME) and Continuing Nursing Education (CNE) activities to disclose all relevant financial relationships with commercial interests. This information is disclosed to CME activity participants prior to the start of the educational activity. Boston University School of Medicine has procedures to resolve all conflicts of interest. In addition, faculty members are asked to disclose when any unapproved use of pharmaceuticals and devices is being discussed.

List of faculty and planners

Shoumita Dasgupta, PhD
Course Director
Professor of Medicine, Biomedical Genetics
Boston University School of Medicine
Boston, MA

Blair Stevens, MS, CGC
Course Faculty
Assistant Professor of Obstetrics, Gynecology, and Reproductive Sciences
University of Texas, McGovern Medical School

Emma Vaimberg, BS
Course assistant, reviewer, planning committee
Medical Student
Boston University School of Medicine

Eric Ford, PhD
Course assistant, reviewer, planning committee
Software developer
Story Street Consulting

Planning Committee members: Julie White, MS, CHCP and Jody Walker, MS of Boston University School of Medicine: Barry M. Manuel Continuing Medical Education Office have no relevant financial relationships to disclose.

All faculty and planning committee members have no relevant financial relationships to disclose. All faculty do not plan on discussing unlabeled/investigational uses of a commercial product.

Start Date: TK

End Date: TK

How to Obtain CME Credit

Complete all reading assignments, answer all questions, and pass the post test with a score of 80% or more. When you have successfully passed the test, we will email you with specific instructions about how to download your certificate.

If you have any questions or concerns, please don't hesitate to contact the CME office:
cme@bu.edu | 617-358-5005

Acknowledgments

AAMC Northeast Group on Educational Affairs
National Human Genome Research Institute

Disclaimer

THIS CONTINUING MEDICAL EDUCATION PROGRAM IS INTENDED SOLELY FOR EDUCATIONAL PURPOSES FOR QUALIFIED HEALTH CARE PROFESSIONALS. THIS PROGRAM DOES NOT REPLACE YOUR HOME COUNTRY'S LAWS, REGULATIONS, AND GUIDELINES CONCERNING MEDICAL CARE AND DRUG PRESCRIPTION ACTIVITIES. IN NO EVENT SHALL BOSTON UNIVERSITY BE LIABLE FOR ANY DECISION MADE OR ACTION TAKEN IN RELIANCE ON THE INFORMATION CONTAINED IN THE PROGRAM. IN NO EVENT SHOULD THE INFORMATION CONTAINED IN THE PROGRAM BE USED AS A SUBSTITUTE FOR PROFESSIONAL CARE. NO PHYSICIAN-PATIENT RELATIONSHIP IS BEING ESTABLISHED.

BUSM CME complies with the ADA and the rules and regulations thereof to make reasonable accommodations for our educational activities. If any participant in this educational activity is in need of accommodations, please contact BUSM CME at 617-358-5005.

Continue

Clinical Scenario One

A couple, Mr. and Mrs. J, comes to you after learning that they are expecting a male child. Mrs. J tells you about her family history of Becker Muscular Dystrophy (BMD) and that she knows she is a carrier for the disease. BMD is an X-linked condition that causes progressive skeletal muscle weakness and cardiomyopathy. BMD typically presents in childhood or adolescence, and individuals with BMD have an average lifespan of 40 to 50 years. The phenotype typically presents only with physical aspects to the condition. Your patient is concerned about her risk for passing on BMD, and she asks you to perform genetic testing to determine if the fetus has Becker Muscular Dystrophy.

Continue

A couple, Mr. and Mrs. J, comes to you after learning that they are expecting a male child. Mrs. J tells you about her family history of Becker Muscular Dystrophy (BMD) and that she knows she is a carrier for the disease. BMD is an X-linked condition that causes progressive skeletal muscle weakness and cardiomyopathy. BMD typically presents in childhood or adolescence, and individuals with BMD have an average lifespan of 40 to 50 years. The phenotype typically presents only with physical aspects to the condition. Your patient is concerned about her risk for passing on BMD, and she asks you to perform genetic testing to determine if the fetus has Becker Muscular Dystrophy.

Select an answer from the choices below

The parents tell you they would terminate the pregnancy, if the fetus were found to have BMD. If you were the patient's healthcare provider, how would you counsel the couple about testing?

- Strongly recommend prenatal testing
- Recommend prenatal testing
- Offer but neither recommend nor discourage prenatal testing
- Discourage prenatal testing
- Strongly discourage prenatal testing

The parents tell you they would NOT terminate the pregnancy if the fetus were found to have BMD. If you were the patient's healthcare provider, how would you counsel the couple about testing?

- Strongly recommend prenatal testing
- Recommend prenatal testing
- Offer but neither recommend nor discourage prenatal testing
- Discourage prenatal testing
- Strongly discourage prenatal testing

If you were personally the patient, would you get prenatal genetic testing for BMD during the pregnancy?

- Yes
- Most likely
- Unsure
- Probably not
- No

If you were personally the patient, if the fetus were found to be affected with BMD, would you seek to terminate the pregnancy?

- Yes
- Most likely
- Unsure
- Probably not
- No

The impact BMD has on physical abilities has had the following influence on my above decisions:

- Great influence
- Moderate influence
- Neutral
- Little influence
- No influence

Submit

Clinical Scenario Two

A 30-year-old woman, Ms. N, comes to you because she recently learned she is a premutation carrier of Fragile X syndrome and is pregnant with a male fetus. Fragile X syndrome is an X-linked condition characterized by cognitive impairment, developmental delays, and autism. She understands that she has a 50% risk to have a baby with Fragile X syndrome and is interested in learning more about her testing options.

Continue

A 30-year-old woman, Ms. N, comes to you because she recently learned she is a premutation carrier of Fragile X syndrome and is pregnant with a male fetus. Fragile X syndrome is an X-linked condition characterized by cognitive impairment, developmental delays, and autism. She understands that she has a 50% risk to have a baby with Fragile X syndrome and is interested in learning more about her testing options.

Select an answer from the choices below

The parents tell you they would terminate the pregnancy, if the fetus were found to have Fragile X syndrome. If you were the patient's healthcare provider, how would you counsel the couple about testing?

- Strongly recommend prenatal testing
- Recommend prenatal testing
- Offer but neither recommend nor discourage prenatal testing
- Discourage prenatal testing
- Strongly discourage prenatal testing

The parents tell you they would NOT terminate the pregnancy, regardless of the results. If you were the patient's healthcare provider, how would you counsel the couple about testing?

- Strongly recommend prenatal testing
- Recommend prenatal testing
- Offer but neither recommend nor discourage prenatal testing
- Discourage prenatal testing
- Strongly discourage prenatal testing

If you were personally the patient, would you get prenatal genetic testing for Fragile X syndrome?

- Yes
- Most likely
- Unsure
- Probably not
- No

If you were personally the patient, would you seek to terminate the pregnancy if the results indicate the fetus has Fragile X syndrome?

- Yes
- Most likely
- Unsure
- Probably not
- No

The impact Fragile X syndrome has on intellectual abilities has had the following influence on my above decisions:

- Great influence
- Moderate influence
- Neutral
- Little influence
- No influence

Submit

Part II of V: Demographics

What is your age?

[ ]

Decline to Answer | Submit

Part II of V: Demographics

What is your current gender identity?

- Male
- Female
- Nonbinary
- Prefer not to answer

Tip: For quick response, click to select your answer, and then click again to submit.

Go Back | Decline to Answer | Submit

Part II of V: Demographics

What is your work setting?

- Clinical laboratory
- Clinical patient-facing
- Research
- Other

Tip: For quick response, click to select your answer, and then click again to submit.

Go Back | Decline to Answer | Submit

Part II of V: Demographics

What is your role in a clinical setting?

- N/A
- MD (medical geneticist)
- MD (other)
- Genetic counselor
- Nurse
- Other allied health professional
- Resident
- Medical student
- Fellow
- GC student
- Educator
- Other

Tip: For quick response, click to select your answer, and then click again to submit.

Go Back | Decline to Answer | Submit

Part II of V: Demographics

What is your state of residence?

-- Choose an option --

Go Back | Decline to Answer | Submit

Part II of V: Demographics

Page 6 out of 13

How religious do you consider yourself to be?

- Very religious
- Somewhat religious
- Not very religious
- Not at all religious

Tip: For quick response, click to select your answer, and then click again to submit.

Go Back | Decline to Answer | Submit

Part II of V: Demographics

Page 7 out of 13

What is your political identity?

- Very Conservative
- Conservative
- Moderate
- Liberal
- Very Liberal
- Other

Tip: For quick response, click to select your answer, and then click again to submit.

Go Back | Decline to Answer | Submit

Part II of V: Demographics

Page 8 out of 13

What is your stance on termination of pregnacy?

- Very Pro Choice (termination is acceptable for any reason)
- Somewhat Pro Choice (termination is acceptable for some reasons)
- Neutral (not pro-choice or pro-life)
- Somewhat Pro-Life (termination is not acceptable except in rare exceptions)
- Very Pro-Life (termination is not acceptable for any reason)

Tip: For quick response, click to select your answer, and then click again to submit.

Go Back | Decline to Answer | Submit

Part II of V: Demographics

Please describe your level of clinical experience counseling pregnant patients about prenatal genetic testing.

- Very experienced
- Somewhat experienced
- Not very experienced
- Not at all experienced

Tip: For quick response, click to select your answer, and then click again to submit.

Go Back | Decline to Answer | Submit

Part II of V: Demographics

Please select relevant experiences you have had with someone with an **intellectual disability (ID)**. Multiple choices are allowed.

- Personal history of ID
- Family member with ID
- Friend with ID
- Volunteer with individuals with ID
- Professional experiences
- Experiences during medical training
- None
- Other

Go Back | Decline to Answer | Submit

Part II of V: Demographics

Page 11 out of 13

Please select relevant experiences you have had with someone with a **physical disability (PD)**. Multiple choices are allowed.

- Personal history of PD
- Family member with PD
- Friend with PD
- Volunteer with individuals with PD
- Professional experiences
- Experiences during medical training
- None
- Other

Go Back | Decline to Answer | Submit

Part II of V: Demographics

Most people are uncomfortable around a child with a *physical disability*.

- Strongly disagree
- Disagree
- Neutral
- Agree
- Strongly agree

The good characteristics of a child with a *physical disability* tend to be ignored.

- Strongly disagree
- Disagree
- Neutral
- Agree
- Strongly agree

Most children with a *physical disability* are extremely impaired and cannot live independently as adults.

- Strongly disagree
- Disagree
- Neutral
- Agree
- Strongly agree

People with *physical disabilities* are often treated as outcasts.

- Strongly disagree
- Disagree
- Neutral
- Agree
- Strongly agree

Most people feel that having a child with a *physical disability* is undesirable.

- Strongly disagree
- Disagree
- Neutral
- Agree
- Strongly agree

Go Back | Submit

Part II of V: Demographics

Most people are uncomfortable around a child with an *intellectual disability*.

- Strongly disagree
- Disagree
- Neutral
- Agree
- Strongly agree

The good characteristics of a child with an *intellectual disability* tend to be ignored.

- Strongly disagree
- Disagree
- Neutral
- Agree
- Strongly agree

Most children with an *intellectual disability* are extremely impaired and cannot live independently as adults.

- Strongly disagree
- Disagree
- Neutral
- Agree
- Strongly agree

People with *intellectual disabilities* are often treated as outcasts.

- Strongly disagree
- Disagree
- Neutral
- Agree
- Strongly agree

Most people feel that having a child with an *intellectual disability* is undesirable.

- Strongly disagree
- Disagree
- Neutral
- Agree
- Strongly agree

Go Back Submit

Next, you will use the 'E' and 'I' computer keys to categorize items into groups as fast as you can. These are the four groups and the items that belong to each:

Category	Items
**Good**	Magnificent, Cherish, Friendship, Fabulous, Fantastic, Lovely, Attractive, Friend
**Bad**	Angry, Yucky, Disaster, Hate, Bothersome, Horrible, Poison, Abuse
**Abled**	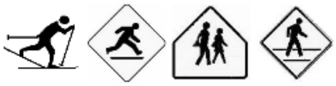
**Disabled**	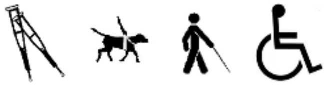

There are seven parts. The instructions change for each part. Pay attention!

Click Here to Proceed

Press "E" for

Physically disabled

Press "I" for

Abled persons

Part 1 of 7

Put a left finger on the **E** key for items that belong to the category Physically disabled.
Put a right finger on the **I** key for items that belong to the category Abled persons.
Items will appear one at a time.

If you make a mistake, a red **X** will appear. Press the other key to continue.
Go as fast as you can while being accurate.

Press the **space bar** when you are ready to start.

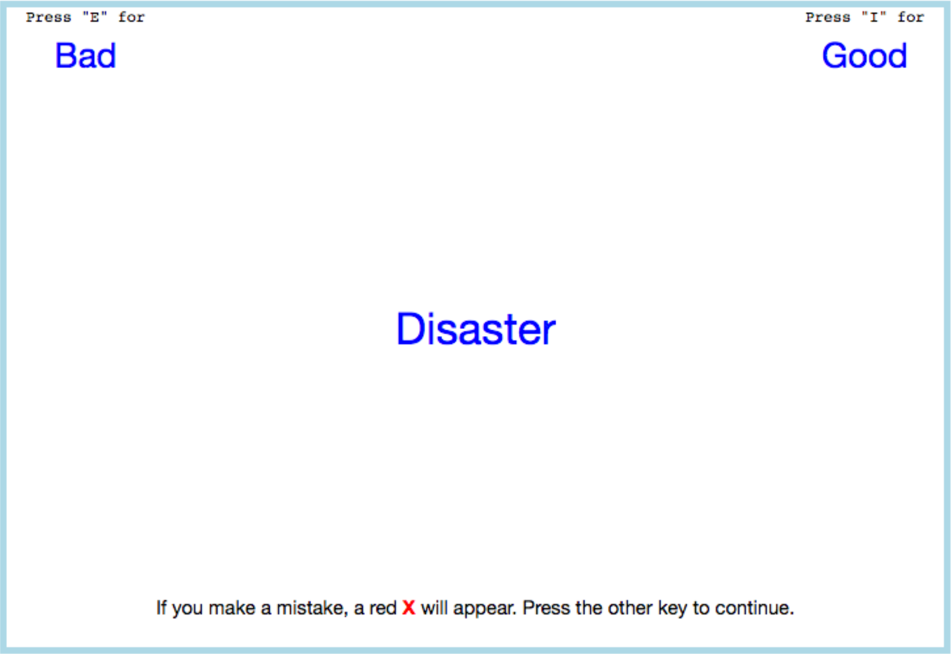
Press "E" for
Bad
Press "I" for
Good
Disaster
If you make a mistake, a red X will appear. Press the other key to continue.

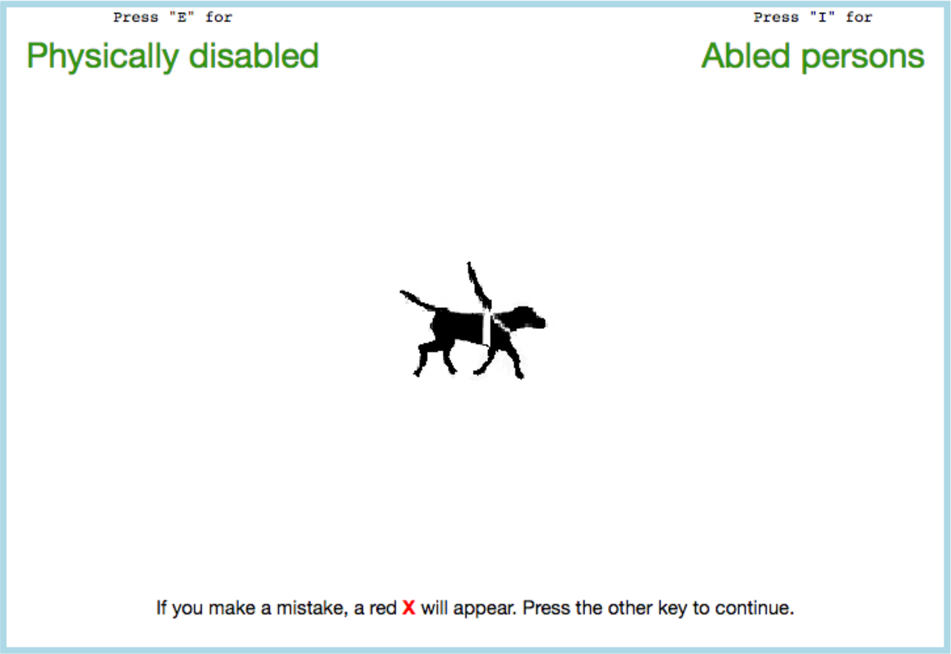
Press "E" for
Physically disabled
Press "I" for
Abled persons
If you make a mistake, a red X will appear. Press the other key to continue.

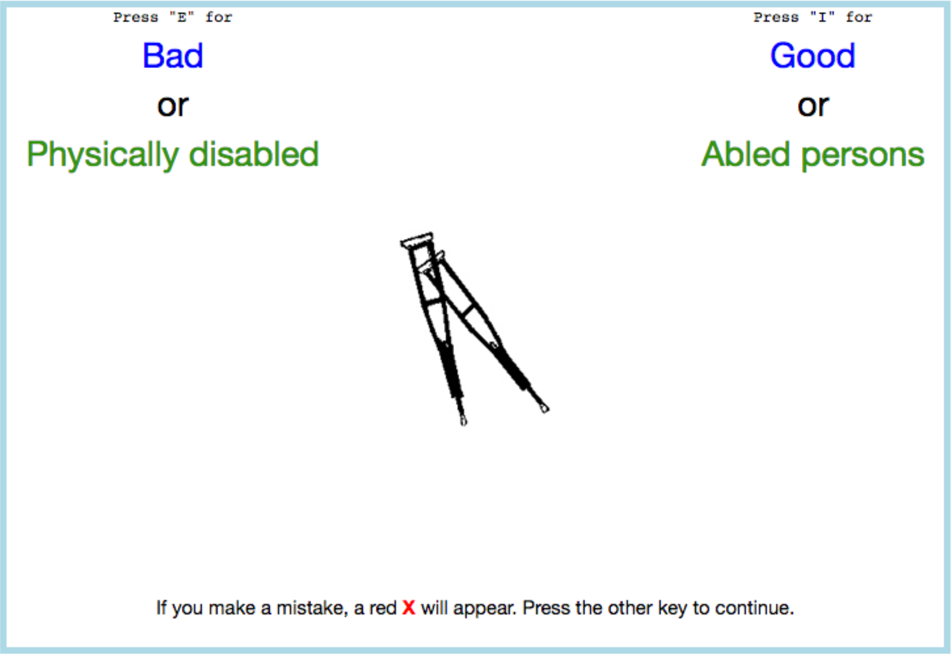
Press "E" for
Bad
or
Physically disabled
Press "I" for
Good
or
Abled persons
If you make a mistake, a red X will appear. Press the other key to continue.

Part III of V: Implicit Association Tests

Thank you for completing the first Implicit Association Test (IAT). There is one more short IAT to work through, and similar instructions apply.

Next, you will use the ‘e’ and ‘i’ computer keys to categorize items into groups as fast as you can. These are the three groups and the items that belong to each:

Category	Items
**Good**	Magnificent, Cherish, Friendship, Fabulous, Fantastic, Lovely, Attractive, Friend
**Bad**	Angry, Yucky, Disaster, Hate, Bothersome, Horrible, Poison, Abuse
**Intellectually Disabled**	Slow learner, Impaired, Dependent, Special needs, Mental handicap

There are seven parts. The instructions change for each part. Pay attention!

Click Here to Proceed

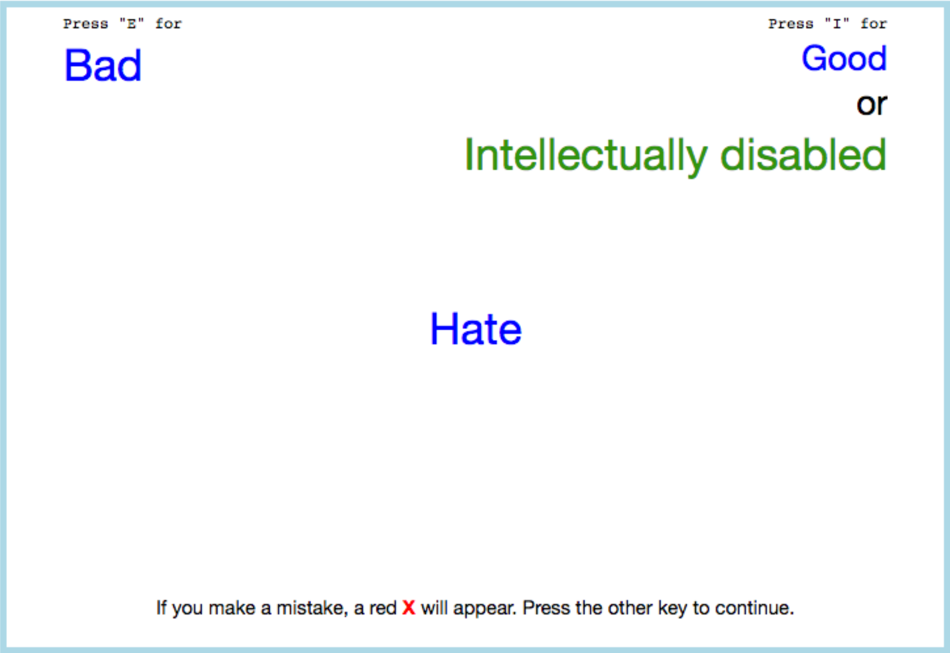
Press "E" for
Bad
Press "I" for
Good
or
Intellectually disabled
Hate
If you make a mistake, a red X will appear. Press the other key to continue.

An Introduction to Patient-Centered Counseling: An Educational Web Module

Introduction

As a medical provider, you will be responsible for helping patients make important decisions about their healthcare. It is not only your responsibility to discuss potential risks and benefits related to medical decisions, but it is also critical for you to understand your patients' goals. Understanding your patients' values can help you advise them on decisions that are consistent with their own wishes and beliefs. Some of the decisions your patients may face can be challenging and may even bring up ethical challenges for both you and your patient. Situations in which you may encounter these challenges could involve risk assessment for hereditary cancer, discussing preconception and reproductive health, or prenatal diagnoses, to name a few.

In prenatal and preconception counseling, there are many important counseling aspects to consider, especially as they relate to prenatal genetic diagnoses and pregnancy management options. In this module, you will learn about patient-centered counseling, patient autonomy, and shared decision-making, and apply these concepts to clinical cases in the prenatal setting.

Goals:

1. Describe a patient-centered model for genetic counseling
2. Outline how to facilitate autonomous decision-making
3. Apply knowledge of patient-centered genetic counseling techniques to clinical case scenarios

Click Here to Proceed

An Introduction to Patient-Centered Counseling: An Educational Web Module

What is Patient-Centered Counseling?

Healthcare providers in a reproductive health setting (both preconception and prenatal) may frequently find themselves educating patients on genetic screening and testing options and delivering test results. Unlike many medical scenarios, the benefits and risks surrounding reproductive decision-making may be uncertain or depend upon patient-specific factors. Therefore, while it is imperative to provide information that is accurate, balanced, and up-to-date, quality patient care is more than just providing information and being an educator. Patient-centered care also includes taking the time to understand the patient's values and how those may be impacted by test results and medical decisions. In other words, a healthcare provider must be an educator, facilitator, advocate, and guide for patients.

As a healthcare provider, you must recognize your own biases and intentions and aim to put yourself in the shoes of your patients so that you can understand their goals and values. When counseling patients, providers should have unconditional positive regard of the patient, empathy, genuineness, and respect for patient autonomy. This approach will empower patients, allowing them to make independent, informed decisions. This is also at the basis of non-directive counseling, which involves helping a patient focus on how to make decisions as opposed to what decision to make.

Key elements of promoting autonomous patient decision-making can include:

1. Building patient self-esteem and competence
2. Assessing prior experience with decision-making
3. Helping the patient structure and understand the decision-making process
4. Exploring reasons for decision-making
5. Exploring differences among stakeholders in the decision
6. Recognizing cultural influences
7. Aiding with feelings of guilt
8. Helping the patient obtain support and guidance from significant others and professionals
9. Helping the patient engage in anticipatory activities

Counselors can facilitate autonomous choice by:

1. Engaging the patient in critical and active reflection
2. Exploring fundamentals of patients' desires
3. Identifying influences on deliberation and decision
4. Evaluating rationality of decision
5. Providing support
6. Not focusing solely on information

Click Here to Proceed

An Introduction to Patient-Centered Counseling: An Educational Web Module

What are some challenges to maintaining a patient-centered approach when counseling patients?

When counseling a patient, you should not be influenced by your personal biases and interests. However, all healthcare providers have personal bias, whether consciously or not, and numerous studies have demonstrated that these biases can have a strong negative impact on patient interactions, including treatments offered and clinical outcomes. Maintaining an empathic connection, free from judgement and coercion, can be challenged by various factors including when a patient has different values or perspectives than the healthcare provider, when assumptions are made about a patient's experience, or when a limited amount of face to face time is available, preventing the development of trust and rapport. When counseling a patient, you can ask yourself:

1. Do you understand the needs of your patient in making decisions?
2. Have you developed effective methods of presenting risk information?
3. Have you explored, respected, and incorporated the patient's personal experiences, beliefs, and attitudes?
4. Have you engaged with the patient in the decision-making process?
5. Have you utilized an interactive style of counseling?

Application of Patient-Centered Counseling

To better understand the patient-centered approach, you will work through a number of cases that could present in a prenatal or pre-conception setting.

A patient/provider scenario will be presented. As the provider, you will be asked to select the response to a patient's question or statement. **You may find there is more than one appropriate response to choose from as there are many effective ways to perform patient-centered counseling.** Click on each response to learn more about each answer option.

Click Here to Proceed

An Introduction to Patient-Centered Counseling: An Educational Web Module

References

1. Chapman, Elizabeth N., Anna Kaatz, and Molly Carnes. “Physicians and implicit bias: how doctors may unwittingly perpetuate health care disparities.” *Journal of general internal medicine* 28.11 (2013): 1504-1510.
2. Cooper, Lisa A., et al. “The associations of clinicians’ implicit attitudes about race with medical visit communication and patient ratings of interpersonal care.” *American journal of public health* 102.5 (2012): 979-987.
3. Green, Alexander R., et al. “Implicit bias among physicians and its prediction of thrombolysis decisions for black and white patients.” *Journal of general internal medicine* 22.9 (2007): 1231-1238.
4. Hoffman, K. M., Trawalter, S., Axt, J. R., & Oliver, M. N. (2016). Racial bias in pain assessment and treatment recommendations, and false beliefs about biological differences between blacks and whites. *Proceedings of the National Academy of Sciences*, 113(16), 4296-4301.
5. Kessler S. Psychological aspects of genetic counseling. XI. Nondirectiveness revisited. *Am J Med Genet* 72:164-171.
6. McCarthy Veach P LB, Callanan N. *Facilitating the Genetic Counseling Process: Practice Based Skills. 2 ed*: Springer International Publishing; 2018.
7. Uhlman W, Schuette J, Yashar B. *Guide to Genetic Counseling*. 2009
8. Weil, Jon, et al. “The relationship of nondirectiveness to genetic counseling: report of a workshop at the 2003 NSGC Annual Education Conference.” *Journal of Genetic Counseling* 15.2 (2006): 85-93.
9. Weil J. *Psychosocial genetic counseling*. New York: Oxford University Press; 2000.

Click Here to Proceed

Case 1

Background: M is pregnant for the first time at 39-years-old after years of infertility. She is 12 weeks along. She reports no personal or family health concerns.

M: I am still in a bit of shock that I am pregnant. I just didn't think I could get pregnant and gave up years ago. But now I'm worried that I am so old, and I've heard of all these risks that increase as you get older. I did a lot of online research, and it looks like there are a bunch of testing options available, right?

Click one of the following statements/questions below to continue the conversation with M:

1. You are correct, let me tell you about the testing options available to you.
2. Yes, there are various genetic tests to choose from. Can you tell me a little more about your concerns, and what test results would mean for you and your pregnancy?
3. Yes, the ideal test for you would be cell-free DNA screening because it is very accurate but doesn't have a risk for miscarriage like invasive tests have.

Case 1

M: A friend from church has a little girl with Down syndrome, and she was one year younger than I am now when she had her. I remember her telling me about how shocked she was when they told her within minutes of delivering that they thought she had Down syndrome. Her daughter is such a sweet, lovely, little girl, but it was very difficult on my friend and her family in the beginning. And I know I would love my child regardless if she had Down syndrome, but I just worry that I won't be here forever, and who will take care of her after I'm gone? So maybe it isn't fair to bring a child into this world that can't take care of herself...

Click one of the following statements/questions below to continue the conversation with M:

1. It sounds like you are somewhat familiar with Down syndrome. Is your friend from church the only exposure to Down syndrome you have had or have you met an adult with Down syndrome?
2. I hear that you are concerned about the wellbeing of your child, but let's first talk about what your chances of Down syndrome are based on your age.
3. It sounds like your friend had a difficult time with the initial diagnosis. Did your friend's experience of finding out her baby has Down syndrome after birth make you realize that you would want to know before delivery if your baby has a genetic condition?

Part IV of V: Patient-Centered Counseling Module

Case 1 of 3

76%

Case 1

M: I feel I am pretty familiar with Down syndrome, and it does worry me, so I think it would be helpful for me to get prenatal testing. I am pretty type A, so I like to be prepared and know as much as I can.

Click one of the following statements/questions below to continue the conversation with M:

1. Based on what you have said, it does sound like you would benefit from testing. In your online research, what have you learned about the various testing options?
2. In order to know as much as possible, I would recommend amniocentesis. It is the most accurate way to test a pregnancy for genetic conditions like Down syndrome.
3. It sounds like you are concerned about Down syndrome. Would you terminate if the results were positive? If the results wouldn't change anything, you may not benefit from genetic testing.

Part IV of V: Patient-Centered Counseling Module

Case 1 of 3

78%

Case 1 Summary:

This case demonstrates how focusing on a patient's needs, desires, and values in the beginning of an appointment can help you tailor information about testing options. By asking questions, we learn that M is familiar with Down syndrome, she saw the impact of a postnatal diagnosis, and it isn't clear whether she would terminate the pregnancy if a genetic condition were identified. This information allows you to tailor information to M's needs and helps you understand motivations behind her testing decisions.

Next Case

Case 2

C and S, both 27-years-old, come to you for preconception counseling. Both of them have achondroplasia due to a heterozygous mutation (one copy) in the FGFR3 gene. Achondroplasia is an autosomal dominant condition associated with short stature (short arms and legs with typical sized trunk), enlarged head, skeletal problems such as lordosis and spinal stenosis, and typical intelligence. They want to discuss with you the chances to have a child who also has achondroplasia. When both parents have achondroplasia, there is a 25% chance the child is unaffected and of typical stature, 50% chance that the child is of short stature with achondroplasia (heterozygous FGFR3 mutation), and a 25% chance that the fetus has a more severe, lethal skeletal dysplasia due to the homozygous (two copies of the FGFR3 mutation) form that typically results in death within the neonatal period due to pulmonary hypoplasia.

	A	a
A	AA<br>Lethal form	Aa<br>Achondroplasia
a	Aa<br>Achondroplasia	aa<br>Typical stature

C: S and I just got married, and we are thinking about starting a family. We understand our condition is genetic, and we are hoping we can have a baby like us.

Click one of the following statements/questions below to continue the conversation with C and S:

1. Congratulations, and I commend you for coming in before you conceive to discuss your concerns. You are correct, achondroplasia is genetic. Do you realize there is also a chance your baby can have a more severe form that can result in severe breathing problems and death?

2. While we all want children to possess our traits, it is not medically recommended to have a child with achondroplasia due to the medical problems associated with the condition. Are you aware that a couple with achondroplasia has a 25% chance of having a healthy child without achondroplasia?

3. You are correct. Achondroplasia is a hereditary condition, and there is a 50% chance you will have a baby with achondroplasia. Can you tell me a little more about what you know about the genetics of achondroplasia and your hopes for your future family?

Case 2

S: We are very aware of all the possibilities. We can have a tall kid, one like us, or the really bad form that causes babies to pass away. But there is a way to test early in pregnancy, right?

Click one of the following statements/questions below to continue the conversation with S:

1. You are correct, S. You both seem well informed. Have you had a chance to discuss what the results of the testing would mean to you and your pregnancy?
2. Absolutely. There is a 25% chance of having a child of typical stature, a 50% chance of achondroplasia, and a 25% chance of the more severe form. Prenatal testing can be performed by invasive testing, but there are some procedure related risks. [Proceed to discussion of risks and benefits of invasive testing vs. screening by ultrasound].
3. Yes, there is a 25% chance the baby can have what is called the “double dominant” form which is usually lethal. Babies may be born alive, but they typically pass soon after birth due to breathing problems.

Case 2

S: My biggest concern is for C. Pregnancy is going to be hard on her body, so we have already had serious discussions about testing and what we would do.

Provider: Can you tell me more about that?

S: We want testing as early as possible because if we find out the baby has the bad form, we do not want to put C through that pregnancy. And to be honest [S looks over at C hesitantly], we haven't really discussed this... but if the results come back showing the baby will be normal sized, I'm thinking it may be best to terminate that pregnancy as well. [Speaking faster now] It's just that C is so small and having a big baby will be even harder for her, and I just can't imagine raising a child that will be taller than me by the time they are in the third grade! It would just be easier to have a baby like us. [C is silent and is looking at the floor]

Click one of the following statements/questions below to continue the conversation with C and S:

1. Thank you for sharing, S. C, how do you feel about what S shared?
2. [To S] It sounds like not only have you been thinking about your future children, but you have also been worried about your wife's health. We can definitely address your concerns related to pregnancy in someone with achondroplasia as well as termination options during pregnancy. Before we go into that, C, how do you feel about what S shared with us?
3. I can understand your concerns, S. Are you familiar with in-vitro fertilization? There is a way you can test embryos before you get pregnant. That way you don't have to terminate a healthy baby.

Case 2 Summary:

In this case, patient-centered counseling techniques and open-ended questions were used to determine the couple's values. In genetic conditions in which extensive modifications to the home or lifestyle are required, parents may elect to have children with the same genetic condition, despite other medical risks that may accompany the desired trait. Other times, the parents may not want a child with the same genetic condition as them. It is important in cases such as this to not make assumptions about what the parents want, support them as they learn more about their options, and help them to understand the risks and benefits.

Next Case

Case 3

S and A, both 30-years-old, come to you because they have recently discovered that S is pregnant. S has a brother, J, with Fragile X syndrome, an X-linked condition characterized by developmental delays, intellectual disabilities, autism, and characteristic facial features. He still lives at home with S's parents.

You take a detailed family history and find out that only J is affected with Fragile X Syndrome. However, S's mother only has one sister, and she does not have any kids. It is likely that S and J's mother is a premutation carrier, which would mean S has a 50% chance of being a carrier herself. If she is a carrier, she has a 50% chance of passing it down.

S grew up taking care of her brother and is conflicted about whether she would want to know before birth if her child had Fragile X.

S: I understand my chances of being a carrier, and part of me wants to get tested so I can be reassured if I come back negative. But, if I am a carrier, I'm not sure I would want to know because then I will just worry.

A: [Frustrated] Yeah, but then we can do more testing to figure out if the baby has it, right?

S: Sure, but that type of testing is risky and may hurt the baby [turns to provider], right?

Click one of the following statements/questions below to continue the conversation with S and A:

1. Yes, the only way to know with certainty whether a baby has Fragile X syndrome is to do invasive testing, such as an amniocentesis. Would you like me to tell you more about the amnio procedure?
2. Yes, additional testing is available, but it is invasive and does have a risk for complications. S, you mentioned feeling conflicted about even wanting to know whether you are a carrier. Tell me more about that.
3. Yes, further testing is available to test the baby, but there is a 50% chance you wouldn't need further testing.

Case 3

I'm just scared that if I end up a carrier, I will worry more, and then if we test the baby and he ends up having Fragile X, I just don't know what I would do. I love my brother very much, and I wouldn't trade him for anything in the world. I don't think I can put myself in the position to have to decide what to do if…[S trails off]. [S turns to provider] What should I do?

[A rolls his eyes because he thinks it is obvious that she should get testing]

Click one of the following statements/questions below to continue the conversation with S and A:

1. There is no right or wrong way to approach testing, but let's explore your options further together. How much have you and A discussed the possibility of raising a child with your brother's condition?
2. There is no right or wrong choice. I support your decision to get testing or to wait until after birth to test the baby. If you want testing, we can draw your blood today. If you need time to think about your options, we can draw your blood at your next appointment.
3. The first step is testing you to determine if you are a carrier. Fragile X carrier screening is safe because it only involves a blood draw. Once we get those results, we can discuss the option of invasive testing in more detail. Hopefully we won't have to worry about it.

Case 3

After thoughtful consideration, S elects to undergo carrier screening, and they plan to undergo prenatal testing if she is a carrier. S is found to carry the premutation (120 CGG repeats), and diagnostic testing confirms the fetus is male with the expanded allele (243 repeats), indicating that the fetus has Fragile X syndrome. The couple returns to you for counseling at 18 weeks.

S: [tearful] I knew this was a possibility, but I was so wishing the testing would tell me my baby is ok. I just don't know if I can be the mother that my mom was to J. I'm not that strong. But I love J so much. I don't know what to do. [A sits silently]

Click one of the following statements/questions below to continue the conversation with S and A:

1. I understand that this was not the result we were hoping for and figuring out what to do now may be one of the most difficult decisions you have had to make. Have you felt comfortable speaking with your family about this?
2. This decision must be very difficult. According to our state law, you have until 22 weeks gestation to make the decision to terminate. I can provide you with the contact information to a clinic, if that is what you choose.
3. Regardless of your decision about the pregnancy, it does not change the love you have for your brother. Can you tell me more about J? What aspects of Fragile X make you most concerned about continuing the pregnancy? A, I would also like to hear your thoughts.

Case 3 Summary:

As a healthcare provider, it is your duty to provide the best care to all of your patients and to do so in a manner that values both the physical and emotional wellbeing of the patient. Providers may feel that they are helping patients by performing certain tests or providing particular treatments. However, if the patient's values are not considered when offering testing, then the testing may, in fact, counter what the patient would actually desire. Using patient-centered counseling techniques can help providers not only facilitate patient decision-making that fits more with their beliefs, but also helps them anticipate results and what emotions and decisions they may face when results are back.

Next

Clinical Scenario Three

A couple, Mr. and Mrs. H, was referred to you for a prenatal counseling session at 12 weeks gestation because they were both found to be carriers of ataxia telangiectasia (AT) through expanded carrier screening ordered by Mrs. H's obstetrician. AT is a condition characterized by childhood onset movement problems called ataxia. They can have difficulty walking and with balance, chorea, and neuropathy. The movement problems typically cause people to require wheelchair assistance by adolescence. They can also have a weakened immune system and an increased risk for cancer. The phenotype typically presents only with physical aspects to the condition. The lifespan varies greatly, but individuals typically survive into early adulthood. When both parents are carriers, each pregnancy has a 25% risk to be affected.

Continue

A couple, Mr. and Mrs. H, was referred to you for a prenatal counseling session at 12 weeks gestation because they were both found to be carriers of ataxia telangiectasia (AT) through expanded carrier screening ordered by Mrs. H's obstetrician. AT is a condition characterized by childhood onset movement problems called ataxia. They can have difficulty walking and with balance, chorea, and neuropathy. The movement problems typically cause people to require wheelchair assistance by adolescence. They can also have a weakened immune system and an increased risk for cancer. The phenotype typically presents only with physical aspects to the condition. The lifespan varies greatly, but individuals typically survive into early adulthood. When both parents are carriers, each pregnancy has a 25% risk to be affected.

Select an answer from the choices below

The couple tells you they would terminate the pregnancy if the fetus was found to have AT. If you were the patient's healthcare provider, how would you counsel the couple about testing?

- Strongly recommend prenatal testing
- Recommend prenatal testing
- Offer but neither recommend nor discourage prenatal testing
- Discourage prenatal testing
- Strongly discourage prenatal testing

The parents tells you they would NOT terminate the pregnancy, regardless of the results. If you were the patient's healthcare provider, how would you counsel the couple about testing?

- Strongly recommend prenatal testing
- Recommend prenatal testing
- Offer but neither recommend nor discourage prenatal testing
- Discourage prenatal testing
- Strongly discourage prenatal testing

If you were personally the patient, would you get prenatal genetic testing for AT?

- Yes
- Most likely
- Unsure
- Probably not
- No

If you were personally the patient, would you seek to terminate the pregnancy based on the results?

- Yes
- Most likely
- Unsure
- Probably not
- No

The impact AT has on physical abilities has had the following influence on my above decisions:

- Great influence
- Moderate influence
- Neutral
- Little influence
- No influence

Submit

Clinical Scenario Four

A 38-year-old female, Ms. F, is referred to you at 18 weeks gestation because she had a prenatal blood test that evaluates DNA from the pregnancy in maternal blood (cell-free DNA screening), and the test has come back indicating the pregnancy has an increased risk to be affected with Down syndrome. Down syndrome is characterized by a wide range of developmental and physical disabilities, including mild to moderate intellectual disabilities, developmental delay, characteristic facial features, congenital heart defects and other congenital anomalies, and an increased risk for several health concerns, most of which are treatable. You counsel the patient that while the blood test results show an increased chance that the fetus has Down Syndrome (approximately 90% chance), it is not definitive, and an amniocentesis is necessary to rule out or diagnose the condition.

Continue

A 38-year-old female, Ms. F, is referred to you at 18 weeks gestation because she had a prenatal blood test that evaluates DNA from the pregnancy in maternal blood (cell-free DNA screening), and the test has come back indicating the pregnancy has an increased risk to be affected with Down syndrome. Down syndrome is characterized by a wide range of developmental and physical disabilities, including mild to moderate intellectual disabilities, developmental delay, characteristic facial features, congenital heart defects and other congenital anomalies, and an increased risk for several health concerns, most of which are treatable. You counsel the patient that while the blood test results show an increased chance that the fetus has Down Syndrome (approximately 90% chance), it is not definitive, and an amniocentesis is necessary to rule out or diagnose the condition.

Select an answer from the choices below

The couple tells you they would terminate the pregnancy if the fetus was found to have Down Syndrome. If you were the patient's healthcare provider, how would you counsel the couple about testing?

- Strongly recommend prenatal testing
- Recommend prenatal testing
- Offer but neither recommend nor discourage prenatal testing
- Discourage prenatal testing
- Strongly discourage prenatal testing

The parents tell you they would NOT terminate the pregnancy, regardless of the results. If you were the patient's healthcare provider, how would you counsel the couple about testing?

- Strongly recommend prenatal testing
- Recommend prenatal testing
- Neither recommend or discourage prenatal testing
- Discourage prenatal testing
- Strongly discourage prenatal testing

If you were personally the patient, would you get prenatal genetic testing for DS?

- Yes
- Most likely
- Unsure
- Probably not
- No

If you were personally the patient, would you seek to terminate the pregnancy based on the results?

- Yes
- Most likely
- Unsure
- Probably not
- No

The impact Downs Syndrome has on intellectual abilities has had the following influence on my above decisions:

- Great influence
- Moderate influence
- Neutral
- Little influence
- No influence

Submit

100%

Thank you for completing the patient-centered counseling module. Our goals in creating this module are four-fold:

1. Uncover biases held by providers/trainees against those with intellectual and/or physical disability
2. Determine how these biases may impact clinical decision-making
3. Design an educational module to teach providers/trainees how to appropriately counsel patients
4. Determine whether an educational intervention has an impact on clinical decision-making

Numerous studies have demonstrated that physician implicit bias can have a negative impact on patient care. We seek to study the impact that an implicit (or unconscious) bias against individuals with intellectual and/or physical disability may have on provider and trainee clinical decision-making. We are particularly focused on the impact this bias could have on counseling in the prenatal setting, as this is an instance in which provider and patient can intervene on pregnancy outcome. As you have experienced, we assessed clinical decision-making through clinical vignettes, followed by our custom Implicit Association Test that measures disability bias. Finally, you have completed an educational module that discusses the principles of patient-centered counseling and shared decision-making, after which we determined the impact of these educational interventions (with or without the patient-centered counseling module) with additional clinical vignettes.

We did not explicitly disclose the focus on provider/trainee biases during the consent process because we felt that disclosing the entire nature of the study would bias the results.

Please feel free to reach out to Dr. Shoumita Dasgupta or Emma Vaimberg with any further questions about this study or if you would like any information on the results.

We are grateful for your help studying the important factors that contribute to clinical guidance.

Continue

About the Implicit Association Test (IAT)

What is the IAT?

The sorting test you took is called the Implicit Association Test (IAT). You first categorized good and bad words with images of Physically disabled, then you categorized good and bad words with labels describing Intellectually disabled.

Here are your physical disability results:

Here are your intellectual disability results:

Interpreting your results

If you were faster responding when *Intellectually disabled* and *Good* are assigned to the same response key than when *Physically disabled* and *Good* were classified with the same key, your result is described as an “Automatic preference for Intellectually disabled over Physically disabled”. Your score is described as an “Automatic preference for Physically disabled over Intellectually disabled” if the opposite occurred.

Your automatic preference may be described as “slight”, “moderate”, “strong”, or “no preference”. This indicates the *strength* of your automatic preference.

The IAT requires a certain number of correct responses in order to get results. If you made too many errors while completing the test you will get the feedback that there were too many errors to determine a result.

Implicit biases are a habit of mind and are frequently in conflict with one’s own values. However, using the patient-centered counseling skills described in this module, you can work to prevent those biases from impacting your patient care.

Note that your IAT result is based only on the categorization task and not on the questions that you answered.

Disclaimer: These results are not a definitive assessment of your implicit preference. The results may be influenced by variables related to the test (e.g., the category labels or particular items used to represent the categories on the IAT) or the person (e.g., how tired you are). The results are provided for educational purposes only.

How Does The IAT Work?

The IAT measures the strength of associations between concepts (e.g., Intellectually disabled, Physically disabled) and attributes (e.g., Good and Bad). The main idea is that making a response is easier when closely related items share the same response key. We would say that one has an implicit preference for Intellectually disabled relative to Physically disabledif they are faster to categorize words when Intellectually disabled and Good share a response key relative to when Intellectually disabled and Bad share a response key.

Any single IAT is unlikely to predict behavior well for a specific individual. In the aggregate, the IAT can predict behavior such as discrimination in hiring and promotion, medical treatment, and decisions related to criminal justice.

Does The Order In Which I Took The IAT Matter?

Yes, the order in which you take the IAT can influence your overall results. But, the effect is very small. So if you first pair Intellectually disabled + Good / Physically disabled + Bad and then pair Physically disabled + Bad / Intellectually disabled + Good, your results might be a just a tiny bit different than they would be if you had done the reverse pairing first. To minimize the order effect, we give more practice trials before the second pairing than we did before the first pairing. We also randomly assign participants to one of the two possible orderings, so half of the test-takers complete Intellectually disabled + Good / Physically disabled + Bad and then Physically disabled + Bad / Intellectually disabled+ Good, and the other half get the opposite order.

I Still Have Questions About The Study.

If you have questions about the IAT, please check out our Frequently Asked Questions (FAQ).

If you have any questions or comments about the study, please email the lead investigator Dr. Shoumita Dasgupta (dasgupta@bu.edu).

Click Here to Proceed

Continuing Education Quiz

Autonomous patient decision making can be promoted by all of the following, EXCEPT:

- Exploring feelings behind decision making
- Asking about prior experiences with decision making
- Disclosing to the patient what you would do if you were him/her
- Building client self esteem

Tip: For quick response, double click on an answer to select it and go to the next page.

Submit

Continuing Education Quiz

What is the best first step to providing patient centered genetic counseling to a pregnant patient who comes to you for prenatal genetic testing?

- Ensuring patient understands that screening blood tests are not 100% accurate
- Exploring the patient's needs, values and desires for information
- Asking the patient whether she would terminate a pregnancy if affected with a genetic condition
- Discussing the timing of when screening and diagnostic testing can be performed

Tip: For quick response, double click on an answer to select it and go to the next page.

Submit

Continuing Education Quiz

Why is exploring patients' desires, needs and values important in genetic counseling?

- Depending on clinical circumstances, genetic testing may be optional.
- The benefits and risks of genetic testing may depend upon unique aspects of an individual.
- Risks and benefits are weighed differently by individuals.
- All of the above

Tip: For quick response, double click on an answer to select it and go to the next page.

Submit

Continuing Education Quiz

A 12-week pregnant patient desires genetic testing because she is 39 years old and is concerned about the increased risk for chromosome abnormalities. What testing would you recommend?

- I would not recommend invasive testing (CVS or amniocentesis) because it will provide the most accurate information.
- I would recommend screening test, such as non-invasive prenatal screening, because it will not pose a risk to her pregnancy.
- I would not recommend any testing to the patient unless she would terminate an affected pregnancy.
- I would first explore the patient's goals for testing in order to help her determine which test, if any, would be helpful to her.

Tip: For quick response, double click on an answer to select it and go to the next page.

Submit

Continuing Education Quiz

Which of the following is true regarding the role implicit bias can play in healthcare?

- It is insurmountable and is always acted upon by the provider.
- It can impact provider-patient relationships.
- It is not relevant to clinical practice.
- It does not impact patient health outcomes.

Tip: For quick response, double click on an answer to select it and go to the next page.

Submit

Continuing Education Quiz

What does the Implicit Association test measure?

- Attitudes and beliefs that people may be unwilling or unable to report
- Conscious attitudes and beliefs about a person or group
- Prejudice or unjust treatment of a person or group
- All of the above

Tip: For quick response, double click on an answer to select it and go to the next page.

Submit

Continuing Education Quiz

Which of the following is not an element of the Implicit Association Test that is used for scoring?

- Reaction time to sorting the words
- Correctly sorting the words
- Providing additional practice when the category associations change
- Scores on explicit bias testing

Tip: For quick response, double click on an answer to select it and go to the next page.

Submit

Continuing Education Quiz

Which of the following is a true statement?

- Implicit Association Test scores predict behavior.
- Implicit Association Tests are only applicable in the setting of genetic prenatal counseling.
- The Implicit Association Test is a validated tool that can promote introspection and possible behavior modification.
- Implicit Association Test scores always correlate with explicit bias.

Tip: For quick response, double click on an answer to select it and go to the next page.

Submit

Your Participation is Important!

Thank you again for participating in this research!

We have learned so much from people like you taking the time to be part of our work.
