# Supplementary material for: Project Inclusive Genetics: Exploring the impact of patient-centered counseling training on physical disability bias in the prenatal setting
Source: PLoS One. 2021 Aug 5;16(8):e0255722. doi: 10.1371/journal.pone.0255722 (PMC8341652; doi:10.1371/journal.pone.0255722)
Supplement: S1 Table — Input data for non-parametric sign test: Sub-grouping of biased for/neutral towards (a) and biased against (b) individuals with PD. Input data for changes in recommendations for genetic testing pre- and post- intervention in scenarios where patients would and would not terminate the pregnancy. (DOCX) [file pone.0255722.s004.docx]

**S1 Table.**

1a. Participants biased for or neutral towards PD.

| Scenario 1  (would terminate) | Negative Difference^a^ | 1 |
| --- | --- | --- |
|  | Positive Difference^b^ | 3 |
|  | No Change | 43 |
|  | Total | 47 |
| Scenario 2  (would not terminate) | Negative Difference^a^ | 1 |
|  | Positive Difference^b^ | 3 |
|  | No Change | 43 |
|  | Total | 47 |

1b. Participants biased against PD.

| Scenario 1  (would terminate) | Negative Difference^a^ | 32 |
| --- | --- | --- |
|  | Positive Difference^b^ | 14 |
|  | No Change | 236 |
|  | Total | 282 |
| Scenario 2  (would not terminate) | Negative Difference^a^ | 25 |
|  | Positive Difference^b^ | 6 |
|  | No Change | 251 |
|  | Total | 282 |

^a^ Negative difference: changing response from would recommend to would not recommend testing

^b^ Positive Difference: changing response from would not recommend to would recommend testing
