# Supplementary material for: Project Inclusive Genetics: Exploring the impact of patient-centered counseling training on physical disability bias in the prenatal setting
Source: PLoS One. 2021 Aug 5;16(8):e0255722. doi: 10.1371/journal.pone.0255722 (PMC8341652; doi:10.1371/journal.pone.0255722)
Supplement: S2 Table — Results from logistic regression analyses pre (a, b) and post (c, d) educational module. (DOCX) [file pone.0255722.s005.docx]

**S2 Table**

**2a. Pre-intervention, patient would terminate**

|  | B | S.E. | Wald | df | p | Exp(B) | Probability |
| --- | --- | --- | --- | --- | --- | --- | --- |
| First-hand Exp | 0.279 | 0.252 | 1.225 | 1 | 0.268 | 1.322 | 0.790 |
| **Professional Exp** | **-0.776** | **0.325** | **5.691** | **1** | **0.017** | **0.46** | **0.613** |
| Explicit Bias | 1.213 | 1.269 | 0.914 | 1 | 0.339 | 3.364 | 0.967 |
| Implicit Bias | 0.136 | 0.108 | 1.57 | 1 | 0.21 | 1.145 | 0.759 |
| personally would test^a^ | 0.244 | 0.347 | 0.497 | 1 | 0.481 | 1.277 | 0.782 |
| **personally would terminate^b^** | **0.978** | **0.283** | **11.928** | **1** | **0.001** | **2.66** | **0.935** |
| Constant | -1.771 | 1.126 | 2.472 | 1 | 0.116 | 0.17 | 0.542 |
| Cox & Snell R^2^ = 0.093 | | | | | | | |

2**b. Pre-intervention, patient would not terminate**

|  | B | S.E. | Wald | df | p | Exp(B) | Probability |
| --- | --- | --- | --- | --- | --- | --- | --- |
| First-hand Exp | 0.099 | 0.334 | 0.088 | 1 | 0.767 | 1.104 | 0.751 |
| **Professional Exp** | **-1.277** | **0.36** | **12.588** | **1** | **< 0.001** | **0.279** | **0.569** |
| Explicit Bias | 3.15 | 1.809 | 3.031 | 1 | 0.082 | 23.334 | 1.000 |
| Implicit Bias | 0.174 | 0.158 | 1.225 | 1 | 0.268 | 1.191 | 0.767 |
| personally would test* | 0.71 | 0.538 | 1.744 | 1 | 0.187 | 2.035 | 0.884 |
| personally would terminate** | -0.029 | 0.364 | 0.006 | 1 | 0.937 | 0.972 | 0.726 |
| Constant | -4.367 | 1.653 | 6.977 | 1 | 0.008 | 0.013 | 0.503 |
| Cox & Snell R^2^ = 0.065 | | | | | | | |

2**c. Post-intervention, patient would terminate**

|  | B | S.E. | Wald | df | p | Exp(B) | Probability |
| --- | --- | --- | --- | --- | --- | --- | --- |
| First-hand Exp | -0.116 | 0.242 | 0.23 | 1 | 0.632 | 0.890 | 0.709 |
| Professional Exp | -0.49 | 0.309 | 2.51 | 1 | 0.113 | 0.613 | 0.649 |
| Explicit Bias | 0.75 | 1.203 | 0.389 | 1 | 0.533 | 2.118 | 0.893 |
| Implicit Bias | 0.01 | 0.103 | 0.01 | 1 | 0.92 | 1.01 | 0.733 |
| personally would test^a^ | -0.112 | 0.693 | 0.026 | 1 | 0.871 | 0.894 | 0.710 |
| personally would terminate^b^ | 0.368 | 0.362 | 1.031 | 1 | 0.31 | 1.444 | 0.809 |
| Constant | -0.625 | 1.22 | 0.262 | 1 | 0.609 | 0.535 | 0.631 |
| Cox & Snell R^2^ = 0.14 | | | | | | | |

2**d. Post-intervention, patient would NOT terminate**

|  | B | S.E. | Wald | df | p | Exp(B) | Probability |
| --- | --- | --- | --- | --- | --- | --- | --- |
| First-hand Exp | 0.292 | 0.387 | 0.57 | 1 | 0.45 | 1.339 | 0.792 |
| **Professional Exp** | **-1.098** | **0.425** | **6.692** | **1** | **0.01** | **0.333** | **0.582** |
| Explicit Bias | 1.902 | 1.976 | 0.927 | 1 | 0.336 | 6.70 | 0.999 |
| **Implicit Bias** | **-0.293** | **0.144** | **4.164** | **1** | **0.041** | **0.746** | **0.678** |
| personally would test^a^ | 0.213 | 1.133 | 0.035 | 1 | 0.851 | 1.237 | 0.775 |
| personally would terminate^b^ | -0.426 | 0.5 | 0.724 | 1 | 0.395 | 0.653 | 0.658 |
| Constant | -0.687 | 1.928 | 0.127 | 1 | 0.722 | 0.503 | 0.623 |
| Cox & Snell R^2^ = 0.040 | | | | | | | |

Bolded text indicates factors that increase the likelihood of *recommending* genetic testing to patients who (a, c) would terminate a PD-positive pregnancy and (b, d) would not terminate a PD-positive pregnancy.

^a^ “personally would test” are respondents who would elect to get prenatal genetic testing for their own pregnancy

^b^ ”personally would terminate” are respondents who would to terminate their own PD-positive pregnancy
