# Supplementary material for: Project Inclusive Genetics: Exploring the impact of patient-centered counseling training on physical disability bias in the prenatal setting
Source: PLoS One. 2021 Aug 5;16(8):e0255722. doi: 10.1371/journal.pone.0255722 (PMC8341652; doi:10.1371/journal.pone.0255722)
Supplement: S3 Table — Input data for non-parametric sign test: Sub-grouping of biased for/neutral towards (a) and biased against (b) individuals with PD. Input data for changes in correctness of recommendations pre- and post- intervention in scenarios where patients would and would not terminate the pregnancy. (DOCX) [file pone.0255722.s006.docx]

**S3 Table.**

**3a. Participants biased for or neutral towards PD**

| Scenario 1  (would terminate) | Negative Difference^a^ | 0 |
| --- | --- | --- |
|  | Positive Difference^b^ | 0 |
|  | No Change | 47 |
|  | Total | 47 |
| Scenario 2  (would not terminate) | Negative Difference^a^ | 5 |
|  | Positive Difference^b^ | 1 |
|  | No Change | 41 |
|  | Total | 47 |

**3b. Participants biased against PD**

| Scenario 1  (would terminate) | Negative Difference^a^ | 1 |
| --- | --- | --- |
|  | Positive Difference^b^ | 1 |
|  | No Change | 280 |
|  | Total | 282 |
| Scenario 2  (would not terminate) | Negative Difference^a^ | 10 |
|  | Positive Difference^b^ | 31 |
|  | No Change | 241 |
|  | Total | 282 |

^a^ Negative difference: changing response from correct to incorrect

^b^ Positive Difference: changing response from incorrect to correct
